# Specific peptide conjugation to a therapeutic antibody leads to enhanced therapeutic potency and thermal stability by reduced Fc dynamics

**DOI:** 10.1038/s41598-023-43431-0

**Published:** 2023-10-02

**Authors:** Masato Kiyoshi, Makoto Nakakido, Abdur Rafique, Minoru Tada, Michihiko Aoyama, Yosuke Terao, Satoru Nagatoishi, Hiroko Shibata, Teruhiko Ide, Kouhei Tsumoto, Yuji Ito, Akiko Ishii-Watabe

**Affiliations:** 1 Division of Biological Chemistry and Biologicals, National Institute of Health Sciences, Kawasaki, Kanagawa Japan; 2 Department of Bioengineering, School of Engineering, The University of Tokyo, Bunkyo-ku, Tokyo, Japan; 3 Chemistry Program, Department of Science, Graduate School of Science and Engineering, Kagoshima University, Korimoto, Kagoshima, Japan; 4 Tosoh Corporation, Ayase, Kanagawa Japan; 5 The Institute of Medical Science, The University of Tokyo, Minato-ku, Tokyo, Japan

**Keywords:** Biological techniques, Biophysics, Biotechnology, Drug discovery, Structural biology, Molecular medicine

## Abstract

Antibody–drug conjugates are powerful tools for combatting a wide array of cancers. Drug conjugation to a therapeutic antibody often alters molecular characteristics, such as hydrophobicity and effector function, resulting in quality deterioration. To develop a drug conjugation methodology that maintains the molecular characteristics of the antibody, we engineered a specific peptide for conjugation to the Fc region. We used trastuzumab and the chelator (DOTA) as model antibody and payload, respectively. Interestingly, peptide/DOTA-conjugated trastuzumab exhibited enhanced antibody-dependent cellular cytotoxicity (ADCC) and increased thermal stability. Detailed structural and thermodynamic analysis clarified that the conjugated peptide blocks the Fc dynamics like a “wedge.” We revealed that (1) decreased molecular entropy results in enhanced ADCC, and (2) blockade of Fc denaturation results in increased thermal stability. Thus, we believe that our methodology is superior not only for drug conjugation but also as for reinforcing therapeutic antibodies to enhance ADCC and thermal stability.

## Introduction

Antibody–drug conjugates (ADCs) are the fastest growing biopharmaceutical drugs because they combine the specificity of antibodies and the cytotoxicity of small-molecule drugs. This powerful combination widens the therapeutic window for combating cancer. To date, 11 ADCs have been approved by the U.S. Food and Drug Administration, and more than 100 ADCs are currently undergoing clinical trials worldwide^1–5^. The recent advent of “homing missile therapy” has driven biopharmaceutical scientists to maximize the therapeutic efficacy of ADCs. Many fundamental studies have focused on the molecular design of ADCs. Specifically, diverse methods for conjugation of payloads to antibodies have been intensively developed to obtain more homogeneous, stable, and potent ADCs^1,6,7^.

In general, chemical conjugation technologies used to generate ADCs target natural amino acids of antibodies, such as lysines or cysteines. Numerous studies have shown that the conjugation to these natural amino acids alters antibody characteristics, including structure, surface charge, solubility (hydrophobicity), and affinity toward interacting molecules (antigen, Fcγ receptors (FcγRs), neonatal Fc receptor (FcRn), and complement)^8,9^. In particular, as discussed in several reports, most cytotoxic drugs used for ADCs are hydrophobic, and thus, lower the aqueous solubility of ADCs^1,10,11^. Low solubility causes antibody aggregation and can increase the risk of immunogenicity in patients^8,12^. This trade-off between molecular instability and cytotoxicity limits further ADC development.

Various site-specific conjugation technologies, such as unnatural amino acid conjugation, SMARTag, N-glycan remodeling, and THIOMAB are on the rise^1,13–16^. However, the high complexity of the conjugation process and the physicochemical instability of ADCs remain unresolved. For manufacturing efficacious and safe ADCs, precise process control is required to reduce batch variation and ensure product quality^17–19^.

The aim of this study was to develop a simple and efficient methodology for drug conjugation that maintains the molecular characteristics of ADCs. Previously, Kishimoto et al. described a methodology for chemical conjugation using an affinity peptide (CCAP) to the Fc region of an antibody^20^. Characterization of the conjugated antibody prepared using the CCAP method indicated interesting features, including enhanced antibody-dependent cellular cytotoxicity (ADCC). However, its underlying mechanisms remain unknown. Here, we first modified the peptide to increase its solubility, and then prepared peptide- and peptide-payload-conjugated antibodies. Subsequently, we evaluated their molecular characteristics in detail, and clarified the underlying molecular mechanisms.

## Results

### Peptide conjugation to trastuzumab

**Figure 1:**
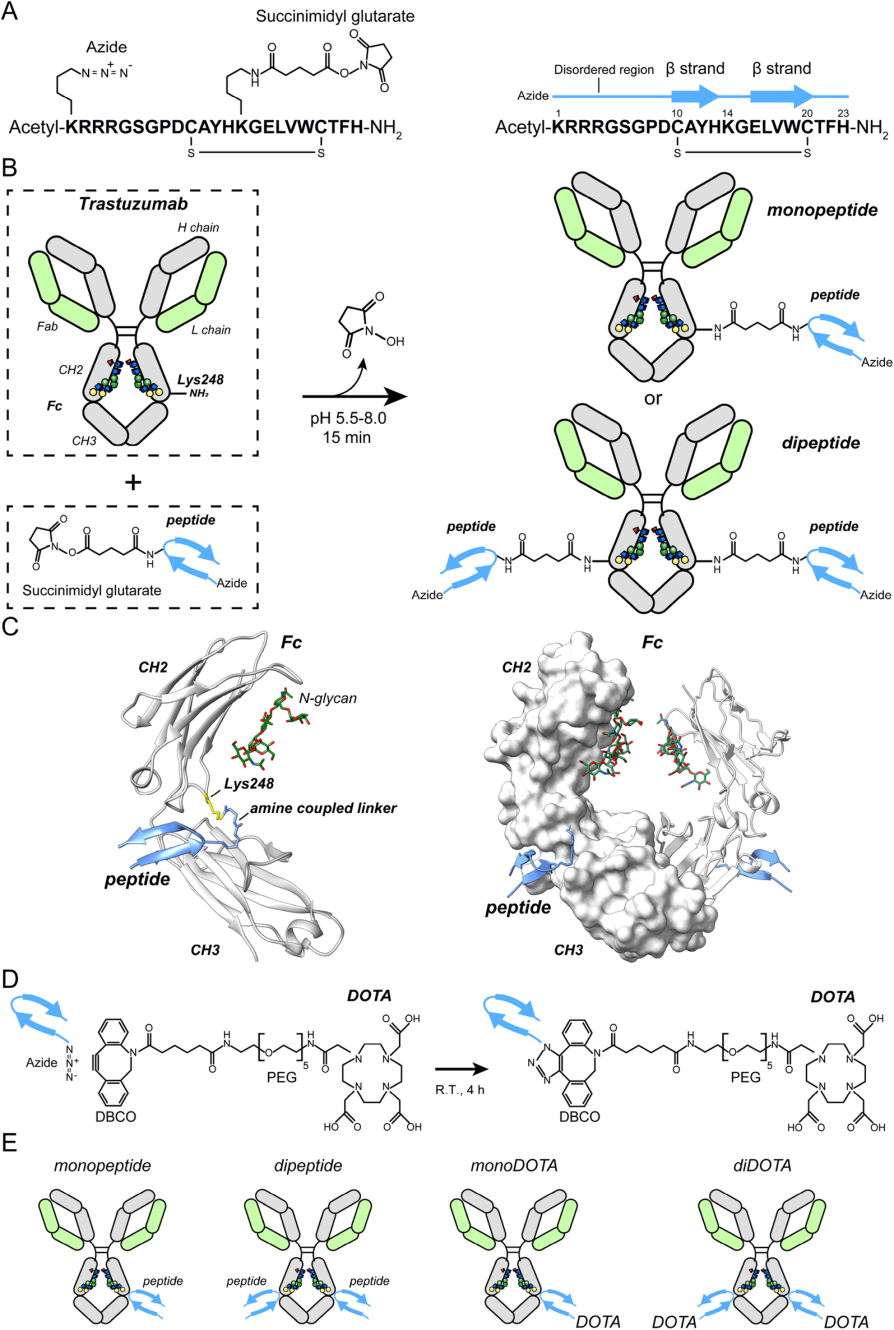
Schematic of the construction of the peptide/DOTA-conjugated trastuzumab. (**A**) Amino acid sequence and chemical modification of the peptide (left). Acetyl-Lys1 was modified with an azide. Lys14 was modified with N-succinimidyl glutarate. The disulfide bond between Cys10 and Cys20 is represented. Secondary structure of the peptide (right). The blue line denotes disordered region. The blue arrows denote β-strands. The sequence numbering is shown. (**B**) The peptide conjugation to the antibody via amine coupling. Stereographic display of N-glycans. Blue cube, green sphere, yellow sphere, and red pyramid correspond to GlcNAc, Man, Gal, and Fuc, respectively. (**C**) Structure of Fc and the previously reported peptide (GPDCAYHKGELVWCTFH) complex (PDB ID, 6IQH). (**D**) DOTA conjugation to the azide via a click reaction. (**E**) Structures of the prepared peptide/DOTA-conjugated trastuzumab.

A 23 amino acid peptide, acetyl-(Lys[Azide]) RRRGSGPDCAYHKGELVWCTFH-NH_2_, was chemically synthesized and modified with a succinimidyl glutarate linker at Lys14 (the numbering starts with Lys[Azide]) (Fig. 1A). To allow payload conjugation and increase the solubility of the conjugates, the N-terminus of the peptide was extended based on a previously reported peptide (GPDCAYHKGELVWCTFH) (referred to as “the shorter peptide”)^20^. Trastuzumab (anti-HER2 humanized IgG1) was used as the model antibody. The amine coupling reaction between the succinimide group of the peptide and the amine of Lys248 (all the antibody residues in this paper are numbered according to EU numbering^21^) in trastuzumab proceeded for 15 min at 25 °C (Fig. 1B). After the conjugation reaction, monovalent peptide-conjugated and divalent peptide-conjugated trastuzumab (referred to as “monopeptide” and “dipeptide,” respectively) were separated using ion-exchange chromatography. The purity of samples was confirmed using ion-exchange chromatography and size-exclusion chromatography (SEC) (Fig. S1A,B). The molecular masses of the monopeptide and dipeptide were determined using liquid chromatography/mass spectrometry (LC/MS) (Fig. S1C). As can be observed in the reported crystal structure of the complex of Fc and the shorter peptide, the peptide located at the groove between CH2 and CH3 binds covalently to the Lys248 of Fc (Fig. 1C)^20^.

### DOTA conjugation to the peptide-conjugated trastuzumab

Our primary aim was to conjugate various payloads, such as therapeutic drugs, DNA, radioisotopes, and antibody domains, to the antibody. In the first trial study, we used 1, 4, 7, 10-tetraazacyclododecane-N, Nʹ, Nʹʹ, Nʹʹʹ-tetraacetic acid (DOTA) as a model payload. DOTA can be labeled with several diagnostic and therapeutic radionuclides. The non-chelated DOTA was modified with dibenzocyclooctyne (DBCO) and polyethylene glycol (PEG). The monopeptide or the dipeptide was mixed with DOTA reagent at 25 °C. The azide-lysine residue of the peptide was coupled to the alkyne of DBCO via a click reaction (Fig. 1D,E). After the conjugation reaction, monovalent and divalent DOTA-conjugated trastuzumab (referred to as “monoDOTA” and “diDOTA,” respectively) were separated using ion-exchange chromatography. The purity of samples was confirmed using ion-exchange chromatography and SEC (Fig. S1A,B). The molecular masses of monoDOTA and diDOTA were determined using LC/MS (Fig. S1C).

### Enhanced ADCC by peptide/DOTA conjugation

**Figure 2:**
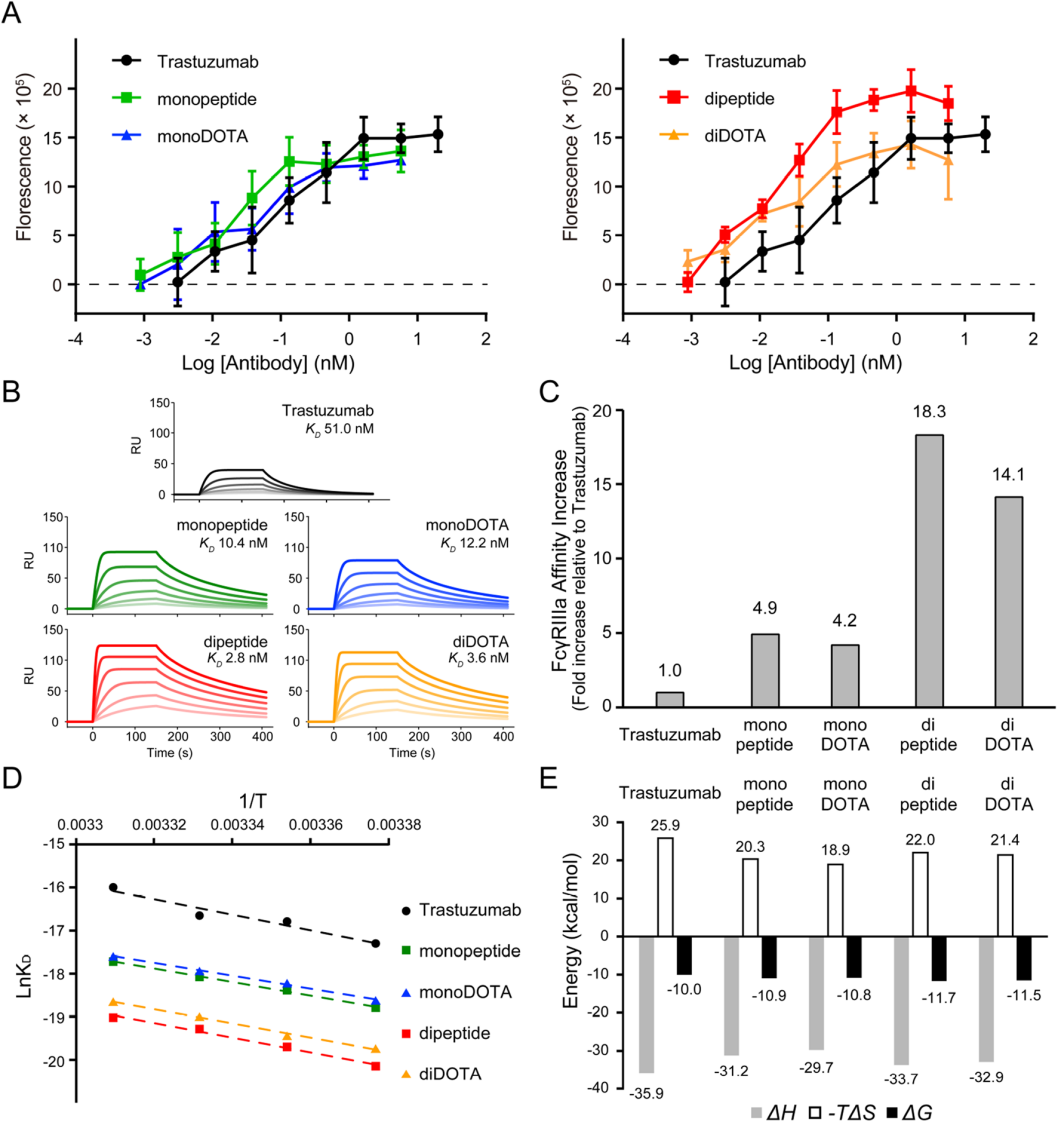
Enhanced ADCC of peptide/DOTA-conjugated trastuzumab. (**A**) ADCC examined using SK-BR-3 cells (target cells) and human PBMCs. Dose–response curves are plotted (trastuzumab, black circle; monopeptide, green square; monoDOTA, blue triangle; dipeptide, red square; diDOTA, orange triangle). (**B**) SPR sensorgrams of FcγRIIIa binding on the sensor chip. Determined affinities (dissociation constant, *K*_D_) are displayed. (**C**) Values of affinity increase relative to trastuzumab. (**D**) The van’t Hoff plot showing the temperature dependence of the affinity. (**E**) Thermodynamic parameters of the binding at 25 °C. (enthalpy change (Δ*H*), gray; entropy change (− *T*Δ*S*), white; Gibbs free energy (Δ*G*), black).

The ADCC of the samples was quantified by measuring cell death of SK-BR-3 cells (HER2-positive cells, target cells) by human peripheral blood mononuclear cells (PBMCs, effector cells). The samples of monopeptide, monoDOTA, dipeptide, and diDOTA showed higher ADCC than trastuzumab (Fig. 2A). The four parameter logistic curve fitting (EC50 shift) was statistically invalid due to both the top and bottom values were changed upon the conjugations. Given that the FcγRIIIa expressed on natural killer (NK) cells in PBMCs plays a pivotal role in ADCC, thus, the affinities of the samples for human FcγRIIIa (CD16a) V158 were determined using surface plasmon resonance (SPR) (Fig. 2B) (Table S1). Trastuzumab bound to FcγRIIIa with a *K*_*D*_ of 51 nM. The affinities of the monopeptide, monoDOTA, dipeptide and diDOTA to FcγRIIIa were remarkably increased, ranging from 4.2- to 18.3-fold, relative to trastuzumab (Fig. 2C). The DOTA conjugation to monopeptide or dipeptide mildly lowers the affinity for FcγRIIIa. Affinity chromatography using the FcγRIIIa-immobilized column also provided evidence for the increased affinity of the peptide/DOTA-conjugated trastuzumab for FcγRIIIa (Fig. S2)^22^. The elution profiles of peptide/DOTA-conjugated trastuzumab were delayed compared with those of trastuzumab.

A van’t Hoff plot was obtained based on the temperature dependence of affinity (Fig. 2D). The thermodynamic parameters of FcγRIIIa binding were calculated from the van’t Hoff plot (Fig. 2E). Trastuzumab bound to FcγRIIIa in an enthalpy-driven manner (Δ*H* = – 35.9 kcal/mol), which was counteracted by an unfavorable entropy loss (*–* *T*Δ*S* = 25.9 kcal/mol). These thermodynamic parameters explain that favorable non-covalent interactions (enthalpy change) overcome the loss of molecular dynamics upon binding (entropy penalty). The values of entropy loss of the monopeptide, monoDOTA, dipeptide, and diDOTA were lower than those of trastuzumab (*–T*Δ*S* = 20.3, 18.9, 22.0, and 21.4 kcal/mol, respectively). The decrease in the entropy loss contributed to the affinity increase, indicating that the molecular dynamics (conformational fluctuations) were lower in the unbound protein state.

### Enhanced ADCC of the peptide-conjugated and afucosylated rituximab

Many studies have shown that the lack of fucose (afucosylation) in N-glycans at Fc enhances ADCC^23–25^. To clarify whether the ADCC enhancement derived from the peptide conjugation can be applied to an afucosyl antibody, we used afucosyl rituximab.

**Figure 3:**
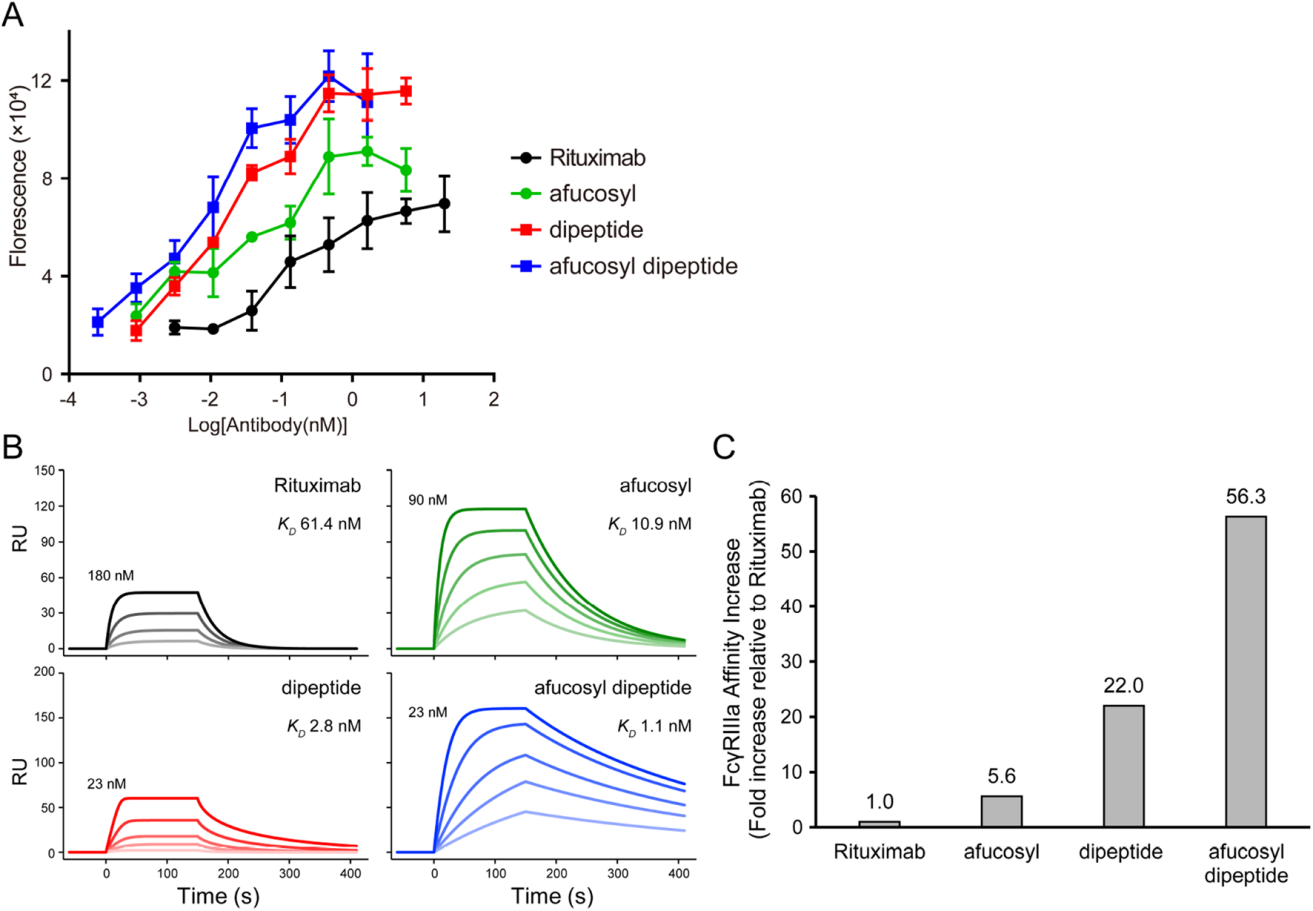
Enhanced ADCC of afucosyl and/or peptide-conjugated rituximab. (**A**) ADCC examined using Raji cells (target cells) and human PBMC. Dose–response curves are plotted (rituximab, black circle; dipeptide, red square; afucosyl, green circle; afucosyl dipeptide, blue square). (**B**) SPR sensorgrams of the FcγRIIIa binding on the sensor chip. Determined affinities (dissociation constant, *K*_D_) are displayed. The highest concentrations in the serial dilution of antibody samples are also displayed. (**C**) Values of affinity increase relative to that of rituximab are displayed.

The afucosyl rituximab was prepared using Fut8 knockout CHO cells (referred to as “afucosyl”). N-glycan analysis showed that the prepared afucosyl contained no detectable fucosylated N-glycans (Fig. S3A,B). The purified afucosyl was subsequently conjugated with the peptide divalently (referred to as “afucosyl dipeptide”). The ADCC of rituximab, dipeptide, afucosyl, and afucosyl dipeptide was quantified by measuring cell death of Raji cells (CD20-positive cells, target cells) by human PBMCs. The samples of dipeptide and afucosyl showed higher ADCC than rituximab (Fig. 3A). Moreover, afucosyl dipeptide, the ADCC of which is enhanced by afucosylation, was further enhanced by the peptide conjugation. The four parameter logistic curve fitting (EC50 shift) was statistically invalid due to the top values were changed upon the conjugations.

The affinities of rituximab, dipeptide, afucosyl, and afucosyl dipeptide for human FcγRIIIa (CD16a) V158 were determined using SPR (Fig. 3B) (Table S1). The unconjugated rituximab bound to FcγRIIIa with a *K*_*D*_ of 61.4 nM. The affinities of the dipeptide, afucosyl, and afucosyl dipeptide were increased relative to rituximab by 5.6-, 22.0-, and 56.3-fold, respectively (Fig. 3C). These results clearly showed that the ADCC enhancement derived from peptide conjugation is applicable to an afucosyl antibodies.

### Structural perspectives of the peptide/DOTA-conjugated antibody Fc

**Figure 4:**
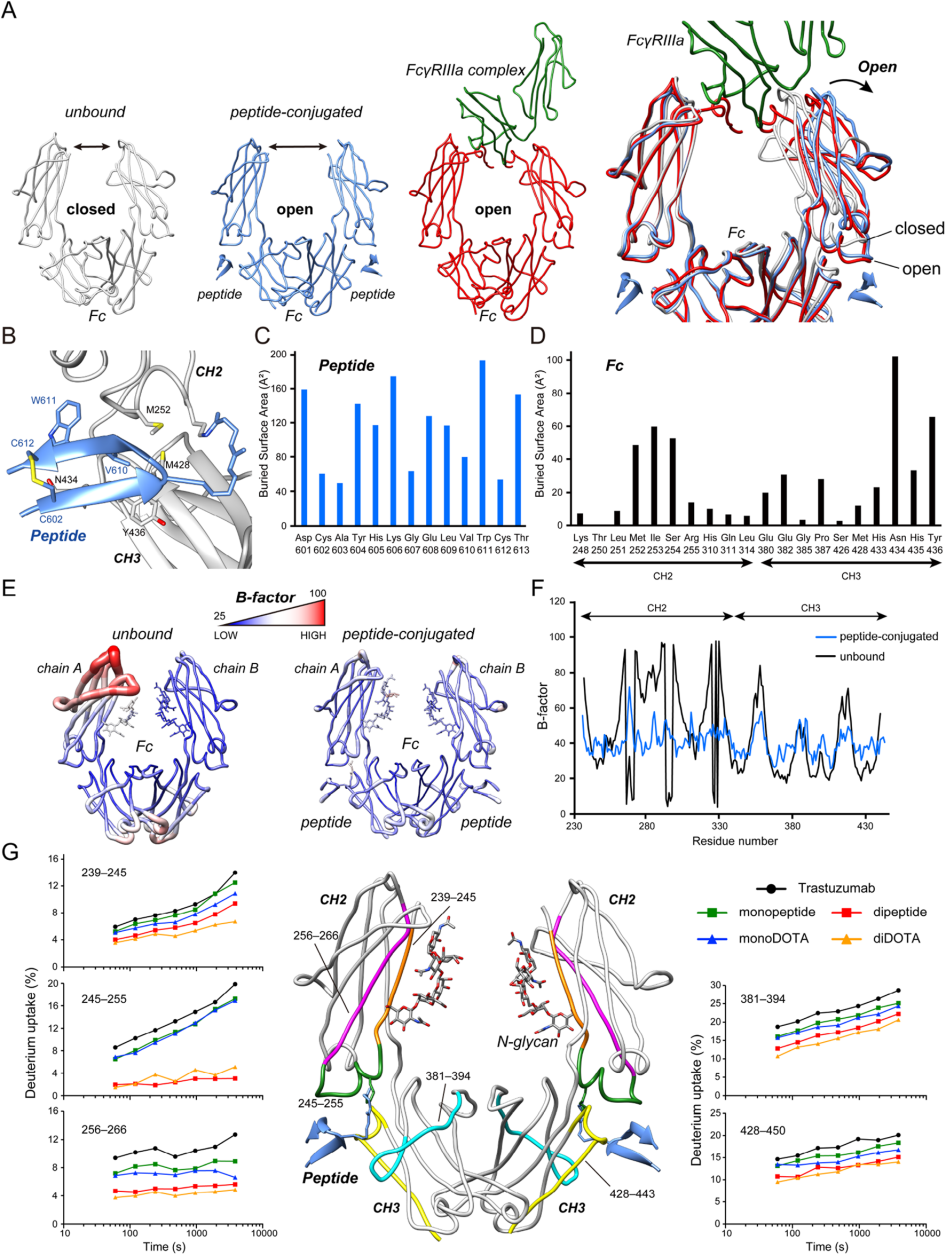
Structural perspectives of peptide/DOTA-conjugated trastuzumab Fc. (**A**) Three Fc structures—unbound (PDB ID 4W4N, white), peptide-conjugated (PDB ID, 6IQH) (blue), and FcγRIIIa-complex (PDB ID 3SGJ) (Fc, red; FcγRIIIa, green)—are shown. (**B**) A focused structural view of peptide-Fc interactions is represented. Peptide, blue; Fc, white; sulfur atoms, yellow; oxygen atoms, red. PDB ID 6IQH. (**C**) Buried surface area (BSA) of each peptide residue. The BSA values were calculated using the PISA server. (**D**) The buried surface area (BSA) of each Fc residue. (**E**) B-factor values in the crystal structure of unbound Fc (PDB ID 4W4N) (left), and peptide-conjugated Fc (PDB ID 6IQH) (right). Regions displaying thick and red cylinders indicate high B-factor values relative to narrow and blue regions. (**F**) Crystallographic B-factors of Fc (4W4N, blue; 6IQH, black). (**G**) HDX analysis of peptide/DOTA-conjugated trastuzumab. The deuterium uptake curves of specific peptides are shown. Ser239–Pro245, orange; Pro245–Arg255, green; Thr256–Val266, magenta; Trp381–Thr394, cyan; Met428–Leu443, yellow.

Attempting to elucidate the fundamental mechanism whereby the ADCC is enhanced, the reported crystal structure of the complex of Fc and the shorter peptide was analyzed in detail^20^. A comparison between the crystal structures of the unbound Fc, peptide-conjugated Fc, Fc-FcγRIIIa complex, and superposition of the structures are shown (Fig. 4A). As discussed in the literature, the Fc structure opens when an antibody binds to FcγRIIIa^26–28^. The structure of the peptide-conjugated Fc is open and quite close to the FcγRIIIa-bound structure. The root-mean-square deviation (RMSD) value between the peptide-conjugated Fc and the FcγRIIIa-complex Fc is only 0.795 Å. Thus, the peptide conjugation induces Fc to be in a prepared (optimized) state for FcγRIIIa-binding.

A closer look at the crystal structure helps us to understand the effects of peptide conjugation. The major interacting residues between the peptide and Fc are illustrated (Fig. 4B). The calculated values of the buried surface area (BSA) of the interacting residues are plotted (Fig. 4C,D). Highly hydrophobic residues of the peptides, such as Val610 and Trp611 (numbering according to the PDB structure), were found to interact with the hydrophobic residues of Fc, such as Met252, Ile253, Met428, and Tyr436. A hydrogen bond between Thr613 of the peptide and Asn434 of Fc also contributes to the strong binding. An internal disulfide bond between Cys602 and Cys612 contributes to peptide stabilization. It should be emphasized that the peptide strongly interacts with the hydrophobic region formed cooperatively by the residues from both the CH2 and CH3 domains.

Multiple studies have described that the Fc structure is in conformational ensembles in aqueous solution^28–31^. The local dynamic behavior of a protein can also be interpreted based on the B-factor or the temperature factor of the crystal structure. The amplitudes of the B-factor of the crystal structures of the unbound Fc and of the complex of Fc and the shorter peptide are displayed (Fig. 4E,F). Although a definite comparison of the B-factor between these two structures is not possible because this factor is intrinsic to the crystal structure, it is safe to assume that the conjugated peptide restricts the structural variation in the CH2 domain of Fc.

Hydrogen deuterium exchange mass spectrometry (HDX-MS) analysis was employed to assess the local antibody dynamics in solution. The deuterium uptake curves of five peptides, ranging from Ser239 to Pro245, Pro245 to Arg255, Thr256 to Val266, Trp381 to Thr394, and Met428 to Leu443 are plotted (Fig. 4G) (Table S2). The deuterium uptake of peptide/DOTA-conjugated trastuzumab was lower than that of trastuzumab, indicating that the fluctuation of Fc was decreased. Moreover, divalent conjugation strengthened this effect. Of note, the deuterium uptake of the peptide ranging from Pro245 to Arg255 was remarkably decreased upon peptide/DOTA conjugation because the Lys248 residue was directly peptide-conjugated.

### Increased thermal stability by peptide/DOTA conjugation

**Figure 5:**
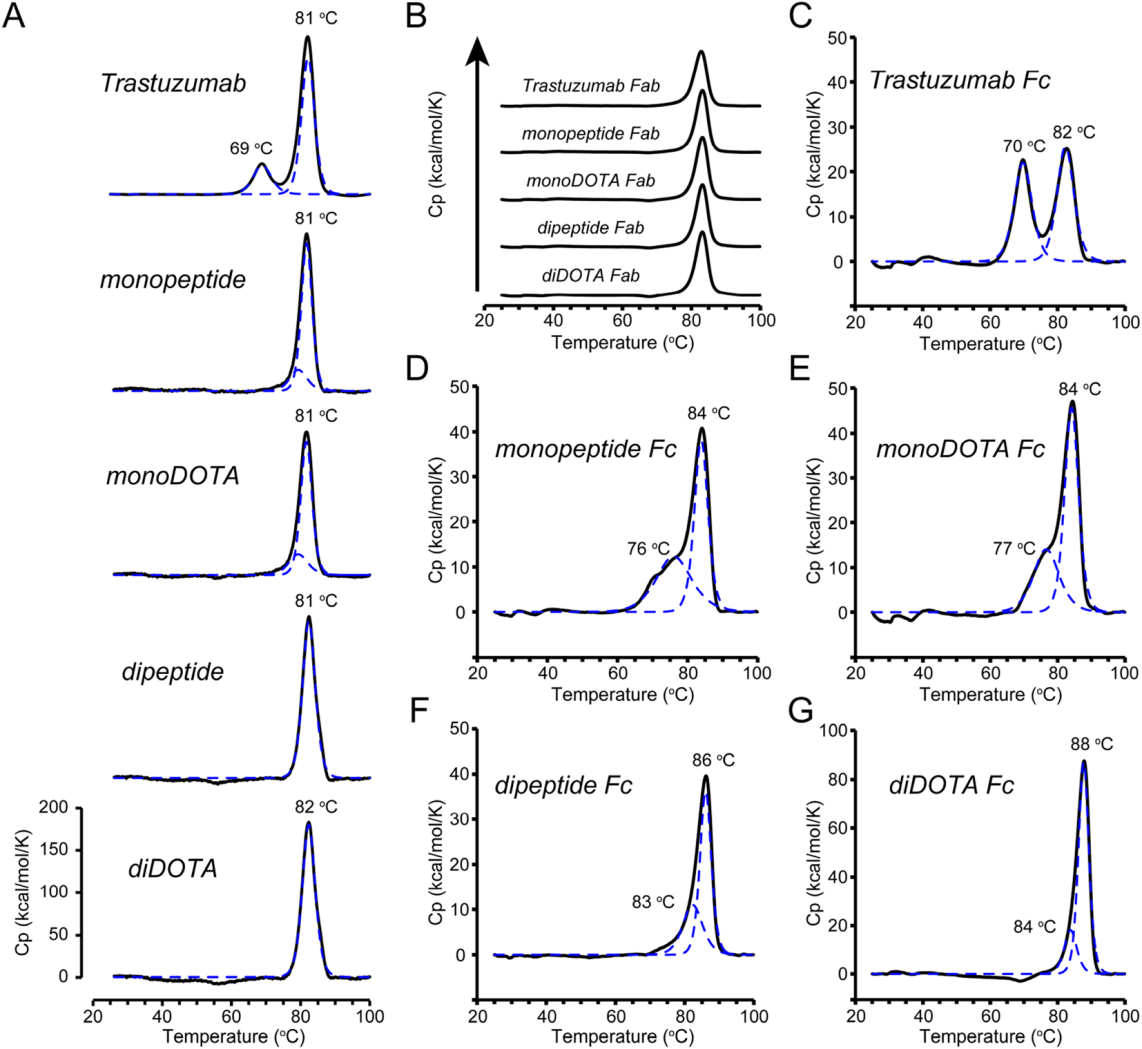
DSC thermograms of full length, Fab and peptide/DOTA-conjugated Fc. (**A**) Thermograms of full length. (**B**) Thermograms of purified Fab showing denaturation peaks at 83 °C. (**C**–**G**) Thermograms of Fc derived from trastuzumab (**C**), monopeptide (**D**), monoDOTA (**E**), dipeptide (**F**), and diDOTA (**G**). Measured DSC thermograms, black solid lines; model fitted curve, blue dotted line. The determined *Tm* values are shown at the top.

Differential scanning calorimetry (DSC) was used to analyze the thermal stability of peptide/DOTA-conjugated trastuzumab. The denaturation thermograms of full-length, Fab, and Fc are represented (Fig. 5A–G). The detailed DSC parameters (*Tonset, Tm*, *ΔH*) are shown (Table S3). For trastuzumab Fc, two denaturation peaks were detected at 70 °C and 82 °C, corresponding to the denaturation of the CH2 and CH3 domains, respectively. The CH2 denaturation peak was decreased, whereas the second peak was increased for monopeptide/monoDOTA Fc, and even more so for the dipeptide/diDOTA Fc. The *Tm* value of the diDOTA Fc was remarkably high (88 °C).

## Discussion

ADCs are the fastest growing biopharmaceutical drugs because they embrace the specificity of antibodies and the cytotoxicity of small-molecule drugs. However, drug conjugations often deteriorate the molecular characteristics of ADCs. It is still challenging to develop ADCs by using efficient and manageable method. These hurdles limit further ADC development.

Here, we sought to develop a simple and efficient methodology for drug conjugation that maintains the molecular characteristics of ADCs. In this method, three components—An antibody, peptides, and payloads—are conjugated in two chemical steps. In the first conjugation, the peptide and antibody are rapidly conjugated under mild conditions. Through the second conjugation, the payloads are coupled to the peptide via a click reaction. The peptide conjugation method has a series of advantages. Various payloads can be conjugated to antibodies, and their number can be controlled. Since the conjugation reaction is highly site-specific, and proceeds spontaneously, the structural modifications (such as reducing disulfide bonds) of the antibody are not necessary. Thus, the conjugation reaction produced a few byproducts. Cleavable linkers, such as peptide linkers, or disulfide linkers, which are often used to design ADCs, undergo deconjugation in the serum, resulting in off-target cytotoxicity^32^. In contrast, the amine coupled linker used in this study was non-cleavable and highly stable^33^. This conjugation method will allow the development of highly efficient and manageable ADCs.

The peptide/DOTA-conjugated trastuzumab exhibited enhanced ADCC compared to the non-conjugated trastuzumab. The decreased molecular dynamics (entropy) of the conjugated trastuzumab contributed to the enhanced affinity for FcγRIIIa. The mechanism underlying this effect is that the conjugated peptide induces Fc to be in a pre-optimized (well-prepared) state for the FcγRIIIa binding, resulting in a thermodynamically favorable binding.

The ADCC enhancement due to the peptide conjugation did not interfere with the effect of afucosylation. The mechanism whereby the affinity of afucosyl antibody for FcγRIIIa is increased has been intensively studied. Multiple structural studies have shown that the N-glycan at Asn162 in FcγRIIIa and the orientation of Tyr296 in Fc are important for this effect^24,25,30,34^. The conjugated peptide was structurally distant from both the N-glycan in FcγRIIIa and Tyr296 in Fc. Therefore, it is reasonable to assume that the peptide conjugation and afucosylation are independently effective. In addition to the afucosylation, multiple bioengineering methodologies have been developed to enhance the therapeutic efficacy, such as mutations (GASDALIE)^35^, ADCs^1^, and bispecific antibodies^36^. This “molecular dynamics-based antibody engineering” opens another window for molecular design of therapeutic antibodies.

**Figure 6:**
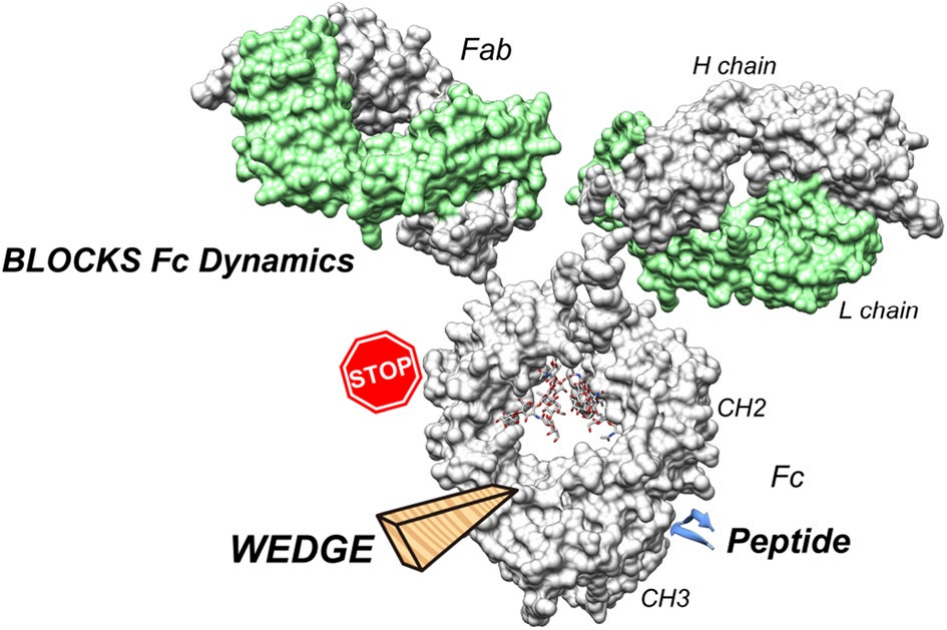
Proposed “wedged antibody” model. The conjugated peptide behaves like a “wedge” that blocks the Fc dynamics, leading to enhanced ADCC and high thermal stability.

Studies using various techniques, such as nuclear magnetic resonance, small-angle X-ray scattering, transmission electron microscopy, and molecular dynamics simulations, have shown that the Fc/antibody structure is in a dynamic ensemble by observing structural variations^28–31,37–41^. The hinge region of the antibody is a pivot of these dynamics^42^. Our previous study revealed that Fc N-glycans modulate these dynamics by interacting with an α-helix (P245–T256) at the interface between CH2 and CH3^22^. Consistent with the previous study, the peptide forms strong interactions with the α-helix and the groove between CH2 and CH3 by acting as a “wedge,” leading to the decreased Fc dynamics (Fig. 6). The findings of these studies show that the α-helix governs the antibody dynamics. The decreased Fc dynamics contributed to the blockade of denaturation, leading to increased thermal stability. The CH2 domain is an aggregation-prone region because of its high flexibility, and the exposure of the hydrophobic residues of CH2 to the solvent is one of the initiating steps for antibody denaturation. Studies on Fc stabilization have been summarized in a review by Yang et al.^43^. The *Tm* of diDOTA Fc was 88 °C, indicating that this is an unprecedented result of Fc stabilization achieved through antibody engineering.

Meanwhile, the affinity of the monopeptide to FcRn was decreased, and the dipeptide showed no binding to FcRn (data not shown), plausibly because the binding sites of both the peptide and FcRn overlap. Accordingly, the decreased affinity of antibodies for FcRn results in a short serum half-life in vivo and in their fast clearance from patients. To achieve the enhanced therapeutic efficacy of the peptide-conjugated antibodies, another method and/or further engineering is required to extend the serum half-life. In the case of radioisotope (RI)-conjugated antibody therapy, the RI-antibody should be eliminated from patients to avoid radiation exposure. Thus, we can choose mono-conjugation or di-conjugation depending on the intended mode of action.

In conclusion, this peptide conjugation methodology is superior not only for drug conjugation but also for reinforcing therapeutic antibodies to enhance ADCC and stability. With further optimization, this technology is expected to be applicable to a broad spectrum of therapeutic antibodies.

## Materials and methods

### Synthesis of the peptides

The peptide (acetyl-(Lys[Azide]) RRRGSGPDCAYHKGELVWCTFH-NH2) was chemically synthesized using the Fmoc solid-phase synthesis method. After removing the protecting group, the intramolecular disulfide bond was formed via oxidation, and the peptide was purified using reverse-phase HPLC. The reaction of the peptide with disuccinimidyl glutarate (DSG) was performed as follows. 41.5 μL of 20 mM peptide in dimethyl sulfoxide (DMSO) containing 1% pyridine was mixed with 16.6 μL of 1 M DSG in DMSO and incubated at 50 °C for 3 h. The reaction product was diluted 250-fold with 0.1% trifluoroacetic acid (TFA) and applied for reverse phase HPLC on InertSustain C18 column (5 μm, 7.6 × 250 mm, GL Sciences) connected to an LC-forte (YMC). The column was eluted using acetonitrile gradient (from 13.5 to 72% both containing 0.1% TFA) for 30 min to isolate the modified peptide reagent which was N-succinimidyl glutarated at the side chain of Lys14. The collected fractions were rapidly evaporated to remove the acetonitrile and lyophilized.

### Peptide conjugation to trastuzumab

0.5 mL of 10 mg/mL trastuzumab solution (68 μM) in PBS was mixed with 3.4 μL of 30 mM peptide reagent at a molar ratio of 1:3, and incubated for 15 min. The samples were diluted with distilled water and subjected to ion-exchange chromatography on IEC SP-825 (8 μm, 8.0 × 75 mm, Shodex) equilibrated with 0.1 M sodium acetate-HCl buffer (pH 5.5). The samples were eluted with a NaCl gradient from 0 to 0.4 M for 30 min at a flow rate of 0.8 mL/min to separate the unmodified, monopeptide and dipeptide. The ion-exchange chromatograms were acquired by monitoring the fluorescence detector at excitation/emission wavelengths of 280/340 nm. After dialysis against the distilled water, the collected fractions were lyophilized. The final yield of the peptide conjugation is approximately 80%. The modification on trastuzumab was confirmed using LC–MS on BioAccord LC–MS System (Waters) connected with Protein BEH C4 Column (2.1 × 5 mm, Waters) which was in a column oven at 80 °C. The elution was done by acetonitrile gradient from 1 to 50% both containing 0.1% formic acid at a flow rate of 0.4 mL/min.

### DOTA conjugation to the peptide-conjugated trastuzumab

The purified monopeptide and dipeptide were reacted with DOTA-PEG5-C6-DBCO (BroadPharm). 1.5 mL of 50 μM monopeptide was mixed with 112.5 μL of 1 mM DOTA-PEG5-C6-DBCO solution in DMSO at a molar ratio of 1:1.5. 1.5 mL of 50 μM dipeptide was mixed with 300 μL of 1 mM DOTA-PEG5-C6-DBCO solution in DMSO at a molar ratio of 1:4. After incubation for 4.5 h at 25 °C, the samples were subjected to the ion-exchange chromatography to remove reagent and unconjugated sample. The purified samples were dialyzed against distilled water and lyophilized. The final yield of the DOTA conjugation is approximately 94%.

### Size exclusion chromatography, SEC

The Superdex 200 10/300 cholumn (Cat. No. 28990944, Cytiva) was used in an ÄKTA Avant system (Cytiva). The mobile phase was 20 mM TrisHCl, 150 mM NaCl at pH 7.4. A total of 0.1 mg of antibody samples was applied to the column at a flow rate of 0.75 mL/min.

### Preparation of rituximab and afucosyl rituximab

Rituximab (Rituxan), an anti CD20 human antibody was purchased from Chugai Pharmaceutical Co., LTD (Japan). The *Fut8* gene of DG44 CHO cells (Invitrogen, USA) was disrupted using the CRISPR Cas9 vector (ORIGENE, USA) according to previously reported methods^44,45^. The cells were incubated at 37 °C with 5% CO_2_ and transfected with the Cas9 vector. Cells were exposed to the screening medium containing *lens culinaris* agglutinin. The selected CHO clone was transfected using expression vector encoding rituximab. Afucosyl rituximab was secreted by cultivation in CD OptiCHO Medium (Cat. No. 12681011, Thermo Fisher Scientific), supplemented with 4% Glutamax (Cat. No. 35050061, Thermo Fisher Scientific), at 37 °C with 5% CO_2_. Recombinant afucosyl rituximab was purified using ÄKTA avant 25 (Cytiva) equipped with a HiTrap Fibro PrismA (Cat. No. 17549855, Cytiva). Subsequently, the afucosyl rituximab was further purified by size-exclusion chromatography using a HiLoad 26/600 Superdex 200 pg column (Cytiva).

### Fc N-glycan analysis

The N-linked oligosaccharides were enzymatically released using PNGase F (Cat. No. 11365169001, Roche). 20 μg of antibody were mixed with 1 U of PNGase F. The mixture was incubated at 37 °C for 16 h, and the resulting digested compounds loaded onto an OASIS HLB cartridge (Cat. No. WAT094225, Waters) pre-treated with 1.0 mL of methanol and 1.0 mL of water. The N-glycan was eluted with 1.0 mL of 10% methanol. The eluted fractions were collected and dried. The labeling solution (10 μL of 0.37 M 2-aminobenzamide (2-AB) and 1 M NaCNBH_3_ in DMSO/acetic acid solution (7/3, v/v)) was added to dried samples of N-glycan. The reaction mixtures were incubated at 65 °C for 3 h. Acetonitrile (1 mL) was gently added to the labeled glycan sample solution, followed by centrifugation at 15,000 × *g* for 10 min. The supernatant was discarded, and the pellet containing the labeled glycan was dissolved in 20 μL of water. A total of 1 μL of glycan solution was subjected to an ACQUITY UPLC BEH amide column (2.1 × 150 mm, 1.7 μm, Waters) in a Waters ACQUITY UPLC H-class system. Mobile phase A was 0.1 M ammonium formate at pH 4.6, and mobile phase B was 100% (v/v) acetonitrile. A linear gradient (from A, 25%; B, 75% to A, 50%; B, 50%) for 50 min was applied and the fluorescence signals were detected at 420 nm (excited at 330 nm). The glycan structures of each peak were determined based on the elution profile.

### Surface plasmon resonance, SPR

The interaction between peptide- and DOTA-conjugated trastuzumab and human FcγRIIIa Val158 was analyzed by SPR in a Biacore 8K instrument (Cytiva). PBS supplemented with 0.005% Tween-20 was used as running buffer. The anti His-tag antibody was first immobilized on the CM5 sensor chip (Cat. No. 29149603, Cytiva). The His-tagged human FcγRIIIa reagent (Cat. No. 10389-H08H1, Sino biologicals) was subsequently injected and captured on the sensor chip. The aimed capture level of FcγRIIIa was 60 RU. As analytes, peptide/DOTA-conjugated trastuzumab serially diluted from 360 to 22.5 nM, were injected. The contact and dissociation time were 150 s and 250 s, respectively. The measurements were conducted at 21 °C, 23 °C, 25 °C, 27 °C, and 29 °C to determine thermodynamic parameters of the FcγRIIIa binding. Data analysis was performed using the evaluation software (Cytiva). The association (*k*_*on*_) and dissociation (*k*_*off*_) rate constants were calculated using a global fitting analysis assuming a Langmuir binding model and a stoichiometry of (1:1). The changes in enthalpy (Δ*H°*) and entropy (Δ*S°*) at 25 °C were calculated from the slope and intercept, respectively, of the temperature dependence of the dissociation constant using the van’t Hoff Eq. (1):

1 $${\text{ln}}K_{D} = \, \Delta H^\circ /RT + \, \Delta S^\circ /R,$$

where *R* is the gas constant and *T* is the absolute temperature.

### The engineered FcγRIIIa-immobilized column chromatography

An engineered FcγRIIIa column chromatography was conducted as described previously^22^. The FcγRIIIa column (Cat. No. 0023532, TOSOH) was used in an ÄKTA Avant system (Cytiva). The mobile phase A was 20 mM sodium acetate, 50 mM NaCl at pH 4.5, and the mobile phase B was 10 mM glycine hydro-chloride at pH 4.5. A total of 1 mg of antibody samples was diluted 20-fold with mobile phase A to adjust the pH to 6.5. A linear gradient of buffer B (0% to 100%) was applied to the column at a flow rate of 0.5 mL/min for 40 min to elute the antibody.

### Antibody dependent cellular cytotoxicity (ADCC) assay

SK-BR-3 cells expressing HER2, and Raji cells expressing CD20 were obtained from the American Type Culture Collection (ATCC, USA) (Cat. No. HTB-30) and Japanese Collection of Research Bioresources (JCRB, Japan) cell bank (Cat. No. JCRB9012), respectively. The cells were cultured in RPMI 1640 medium supplemented with 10% FBS. Human PBMCs were purchased from Cellular Technology Limited (CTL, USA). SK-BR-3 cells at 5 × 10^3^ cells/well were seeded in 96-well plates (Cat. No. 6005181, PerkinElmer). After overnight incubation, human PBMCs at 2 × 10^5^ cells/well were seeded in CTL-Test medium (Cat. No. CTLT-010, CTL) containing Cell Tox Green reagent (Cat. No. G8742, Promega). Serially diluted antibody samples were added. After incubation for 24 h at 37 °C, fluorescence intensity (excitation at 485 nm and emission at 520 nm) was measured using an EnSight plate reader (PerkinElmer, USA). The measurements were performed in triplicate. The dose–response curves were obtained by subtracting the average value of the background wells. The ADCC assays for rituximab were performed using the Raji cells, as described above. A non-linear curve fitting was performed by four parameter logistic model using GraphPad Prism6.

### Structural analysis

The peptide-Fc interacting residues and their BSA values were calculated using the PISA server^46^. The Cα RMSD value was determined using UCSF Chimera v1.16.

### Hydrogen deuterium exchange mass spectrometry, HDX-MS

Trasutuzumab, monopeptide, monoDOTA, dipeptide, and diDOTA were prepared in PBS containing 5% DMSO at a final concentration of 1.0 mg/mL. Each protein was diluted tenfold with PBS containing 5% DMSO in deuterium water (D_2_O). The diluted solutions were then incubated separately at each time point and at 10 °C (Table S2). The deuterium-labeled samples were quenched by diluting them by approximately tenfold with the quenching buffer at pH 3.0, which was composed of 8 M urea and 1 M Tris (2-carboxyethyl) phosphine hydrochloride. All of the processes described above were performed automatically using HDx-3 PAL (LEAP Technologies). After quenching, the solutions were subjected to online pepsin digestion followed by LC/MS analysis using an UltiMate3000RSLCnano (Thermo Fisher Scientific) connected to a Q Exactive plus mass spectrometer (Thermo Fisher Scientific). Online pepsin digestion was performed with a Poroszyme Immobilized Pepsin Cartridge 2.1 × 30 mm (Waters Corporation) in formic acid solution (pH 2.5) for 3 min at 8 °C, at a flow rate of 50 μL/min. Desalting and analytical processes were performed using Acclaim PepMap 300 C18 (1.0 mm × 15 mm, Thermo Fisher Scientific) and Hypersil GOLD (1.0 mm × 50 mm, Thermo Fisher Scientific) columns. The mobile phases were 0.1% formic acid solution (buffer A) and 0.1% formic acid containing 90% acetonitrile (buffer B). The deuterated peptides were eluted at a flow rate of 45 μL/min using a gradient of 10% to 90% of buffer B for 9 min. The conditions of the mass spectrometer were as follows: electrospray voltage, 3.8 kV; positive ion mode, sheath and auxiliary nitrogen flow rate at 20 and two arbitrary units; ion transfer tube temperature at 275 °C; auxiliary gas heater temperature at 100 °C; and a mass range of m/z 200 to 2000. Data-dependent acquisition was performed using a normalized collision energy of 27 arbitrary units. The MS and MS/MS spectra were subjected to a database search analysis using the Proteome Discoverer 2.2 (Thermo Fisher Scientific). Analysis of the deuteration levels of the peptide fragments was performed based on the MS raw files, by comparing the spectra of deuterated samples with those of non-deuterated samples, using the HDExaminer software (Sierra Analytics). Data summary for each HDX-MS is shown (Table S2).

### Differential scanning calorimetry, DSC

The thermal stability of each antibody sample was determined using a PEAQ-DSC instrument (Malvern, UK). The Fab and Fc was separated by papain digestion (#44,985, Thermo Scientific), and purified using an engineered protein A column (Cat. No. KPA02-C001, KanCap, Kaneka, Japan) (Fig. S4). Protein samples were dialyzed against 4 mM Histidine, 150 mM NaCl, pH 6.2 prior to each scan. Protein samples at a concentration of 5 μM were subjected to DSC measurements from 25 to 100 °C at a scan rate of 90 °C/h. The thermograms were normalized by subtracting the signal of the reference cell containing only buffer. The melting temperature (*Tm*) values were calculated by a standard fitting procedure using evaluation software using a non-two state model.

### Supplementary Information

Supplementary Information.

## Data Availability

The data presented in this study is contained within the article and supplementary material. The datasets are available from the corresponding author on reasonable request.

## References

[1] Beck A, Goetsch L, Dumontet C, Corvaia N (2017). Strategies and challenges for the next generation of antibody-drug conjugates. Nat. Rev. Drug Discov..

[2] McCombs JR, Owen SC (2015). Antibody drug conjugates: Design and selection of linker, payload and conjugation chemistry. AAPS J..

[3] Smaglo BG, Aldeghaither D, Weiner LM (2014). The development of immunoconjugates for targeted cancer therapy. Nat. Rev. Clin. Oncol..

[4] Chau CH, Steeg PS, Figg WD (2019). Antibody-drug conjugates for cancer. Lancet.

[5] Pettinato MC (2021). Introduction to antibody-drug conjugates. Antibodies (Basel).

[6] Kline T (2015). Methods to make homogenous antibody drug conjugates. Pharm. Res..

[7] Ekholm FS (2016). Introducing glycolinkers for the functionalization of cytotoxic drugs and applications in antibody-drug conjugation chemistry. ChemMedChem.

[8] Buecheler JW, Winzer M, Weber C, Gieseler H (2020). Alteration of physicochemical properties for antibody-drug conjugates and their impact on stability. J. Pharm. Sci..

[9] Shen BQ (2012). Conjugation site modulates the in vivo stability and therapeutic activity of antibody-drug conjugates. Nat. Biotechnol..

[10] Lyon RP (2015). Reducing hydrophobicity of homogeneous antibody-drug conjugates improves pharmacokinetics and therapeutic index. Nat. Biotechnol..

[11] Burke PJ (2017). Optimization of a PEGylated glucuronide-monomethylauristatin E linker for antibody-drug conjugates. Mol. Cancer Ther..

[12] Duerr C, Friess W (2019). Antibody-drug conjugates-stability and formulation. Eur. J. Pharm. Biopharma. Off. J. Arbeitsgemeinschaft Pharm. Verfahrenstechnik.

[13] Tian F (2014). A general approach to site-specific antibody drug conjugates. Proc. Natl. Acad. Sci. U.S.A..

[14] Drake PM (2014). Aldehyde tag coupled with HIPS chemistry enables the production of ADCs conjugated site-specifically to different antibody regions with distinct in vivo efficacy and PK outcomes. Bioconjug. Chem..

[15] van Geel R (2015). Chemoenzymatic conjugation of toxic payloads to the globally conserved N-glycan of native mAbs provides homogeneous and highly efficacious antibody-drug conjugates. Bioconjug. Chem..

[16] Zacharias N (2022). A homogeneous high-DAR antibody-drug conjugate platform combining THIOMAB antibodies and XTEN polypeptides. Chem. Sci..

[17] Ou J (2018). Bioprocess development of antibody-drug conjugate production for cancer treatment. PloS One.

[18] Hamblett KJ (2004). Effects of drug loading on the antitumor activity of a monoclonal antibody drug conjugate. Clin. Cancer Res. Off. J. Am. Assoc. Cancer Res..

[19] Rathore AS, Winkle H (2009). Quality by design for biopharmaceuticals. Nat. Biotechnol..

[20] Kishimoto S (2019). Site-specific chemical conjugation of antibodies by using affinity peptide for the development of therapeutic antibody format. Bioconjug. Chem..

[21] Edelman GM (1969). The covalent structure of an entire gammaG immunoglobulin molecule. Proc. Natl. Acad. Sci. U.S.A..

[22] Kiyoshi M (2018). Assessing the heterogeneity of the Fc-glycan of a therapeutic antibody using an engineered FcgammaReceptor IIIa-immobilized column. Sci. Rep..

[23] Shields RL (2002). Lack of fucose on human IgG1 N-linked oligosaccharide improves binding to human Fcgamma RIII and antibody-dependent cellular toxicity. J. Biol. Chem..

[24] Ferrara C (2011). Unique carbohydrate-carbohydrate interactions are required for high affinity binding between FcgammaRIII and antibodies lacking core fucose. Proc. Natl. Acad. Sci. U.S.A..

[25] Mizushima T (2011). Structural basis for improved efficacy of therapeutic antibodies on defucosylation of their Fc glycans. Genes Cells Devoted Mol. Cell. Mech..

[26] Pincetic A (2014). Type I and type II Fc receptors regulate innate and adaptive immunity. Nat. Immunol..

[27] Krapp S, Mimura Y, Jefferis R, Huber R, Sondermann P (2003). Structural analysis of human IgG-Fc glycoforms reveals a correlation between glycosylation and structural integrity. J. Mol. Biol..

[28] Caaveiro JM, Kiyoshi M, Tsumoto K (2015). Structural analysis of Fc/FcgammaR complexes: A blueprint for antibody design. Immunol. Rev..

[29] Waibl F (2021). Conformational ensembles of antibodies determine their hydrophobicity. Biophys. J..

[30] Yanaka S (2019). Dynamic views of the Fc region of immunoglobulin G provided by experimental and computational observations. Antibodies (Basel).

[31] Frank M, Walker RC, Lanzilotta WN, Prestegard JH, Barb AW (2014). Immunoglobulin G1 Fc domain motions: Implications for Fc engineering. J. Mol. Biol..

[32] Dere R (2013). PK assays for antibody-drug conjugates: Case study with ado-trastuzumab emtansine. Bioanalysis.

[33] Dorywalska M (2015). Site-dependent degradation of a non-cleavable Auristatin-based linker-payload in rodent plasma and its effect on ADC efficacy. PloS One.

[34] Ferrara C, Stuart F, Sondermann P, Brunker P, Umana P (2006). The carbohydrate at FcgammaRIIIa Asn-162. An element required for high affinity binding to non-fucosylated IgG glycoforms. J. Biol. Chem..

[35] Smith P, DiLillo DJ, Bournazos S, Li F, Ravetch JV (2012). Mouse model recapitulating human Fcgamma receptor structural and functional diversity. Proc. Natl. Acad. Sci. U.S.A..

[36] Labrijn AF, Janmaat ML, Reichert JM, Parren P (2019). Bispecific antibodies: A mechanistic review of the pipeline. Nat. Rev. Drug Discov..

[37] Houde D, Peng Y, Berkowitz SA, Engen JR (2010). Post-translational modifications differentially affect IgG1 conformation and receptor binding. Mol. Cell. Proteom. MCP.

[38] Voynov V (2009). Dynamic fluctuations of protein-carbohydrate interactions promote protein aggregation. PloS One.

[39] Zhang X (2015). 3D structural fluctuation of IgG1 antibody revealed by individual particle electron tomography. Sci. Rep..

[40] Subedi GP, Barb AW (2015). The structural role of antibody N-glycosylation in receptor interactions. Structure.

[41] Kiyoshi M, Tsumoto K, Ishii-Watabe A, Caaveiro JMM (2017). Glycosylation of IgG-Fc: A molecular perspective. Int. Immunol..

[42] Chen Q (2017). Reconstruction of 3D structures of MET antibodies from electron microscopy 2D class averages. PloS One.

[43] Yang C, Gao X, Gong R (2017). Engineering of Fc fragments with optimized physicochemical properties implying improvement of clinical potentials for Fc-based therapeutics. Front. Immunol..

[44] Zong H (2017). Producing defucosylated antibodies with enhanced in vitro antibody-dependent cellular cytotoxicity via FUT8 knockout CHO-S cells. Eng. Life Sci..

[45] Yamane-Ohnuki N (2004). Establishment of FUT8 knockout Chinese hamster ovary cells: An ideal host cell line for producing completely defucosylated antibodies with enhanced antibody-dependent cellular cytotoxicity. Biotechnol. Bioeng..

[46] Krissinel E, Henrick K (2007). Inference of macromolecular assemblies from crystalline state. J. Mol. Biol..

