# Supplementary material for: Specific peptide conjugation to a therapeutic antibody leads to enhanced therapeutic potency and thermal stability by reduced Fc dynamics
Source: Sci Rep. 2023 Oct 2;13:16561. doi: 10.1038/s41598-023-43431-0 (PMC10545826; doi:10.1038/s41598-023-43431-0)
Supplement: Supplementary file 1 — Supplementary Information. [file 41598_2023_43431_MOESM1_ESM.docx]

**Supporting Information for**

**Specific peptide conjugation to a therapeutic antibody leads to enhanced therapeutic potency and thermal stability by reduced Fc dynamics**

Masato Kiyoshi, Makoto Nakakido, Abdur Rafique, Minoru Tada, Michihiko Aoyama, Yosuke Terao, Satoru Nagatoishi, Hiroko Shibata, Teruhiko Ide, Kouhei Tsumoto, Yuji Ito, and Akiko Ishii-Watabe

Corresponding author: Masato Kiyoshi and Yuji Ito

Email: m.kiyoshi@nihs.go.jp or yito@sci.kagoshima-u.ac.jp


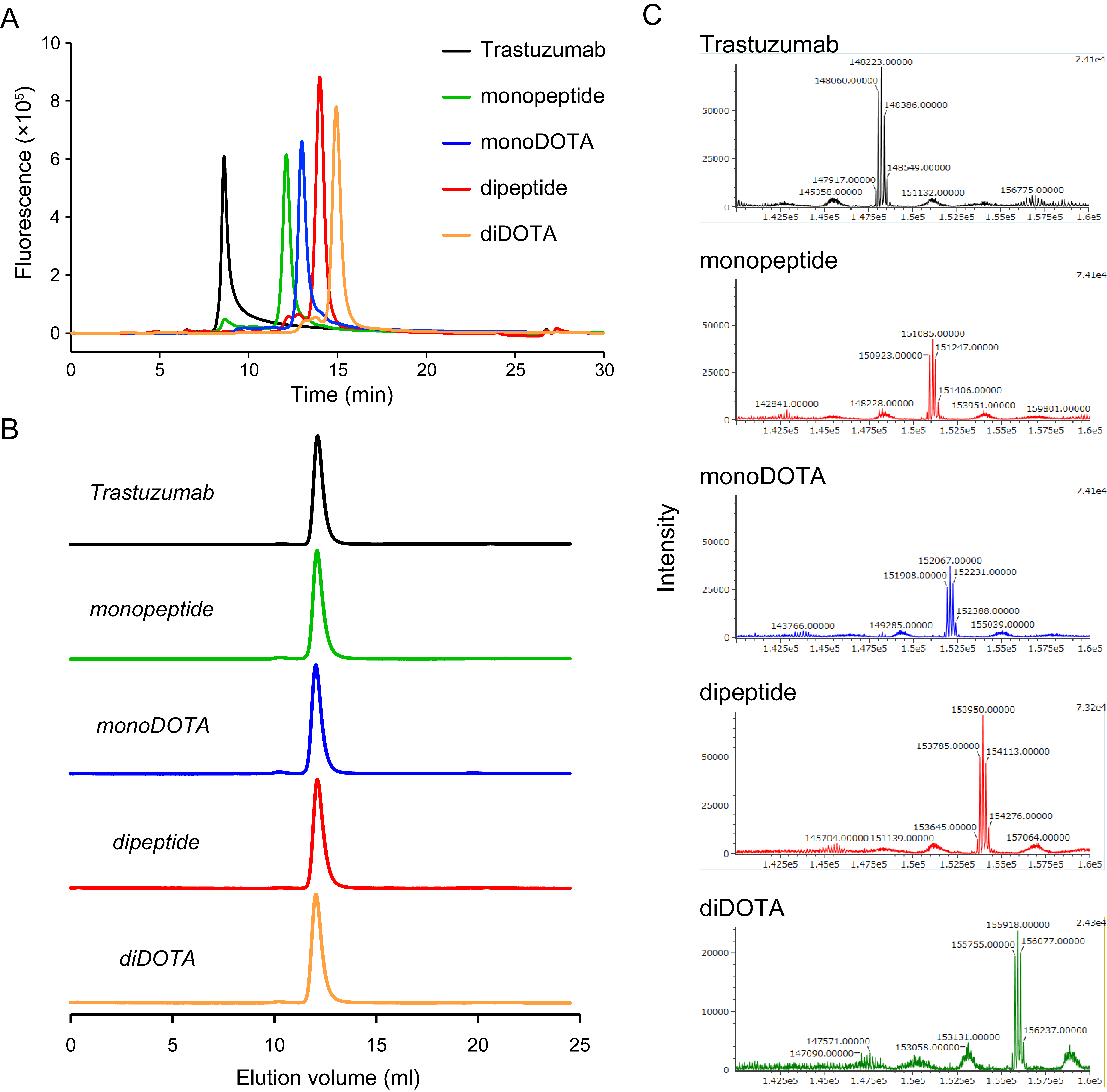


Fig. S1. Preparation of peptide/DOTA-conjugated trastuzumab.

(A) The Cation exchange chromatography of the samples. (B) The SEC profiles of purified samples. (C) Deconvoluted ESI mass spectra. The measured mass of the trastuzumab (G0F) is 148,060. The mass difference between monopeptide and trastuzumab (150,923 – 148,060 = 2,863) corresponds to the peptide conjugation. The mass difference between monoDOTA and monopeptide (151,908 – 150,923 = 985) corresponds to the DBCO-PEG-DOTA conjugation. The divalent conjugations of peptide/DOTA were also confirmed.


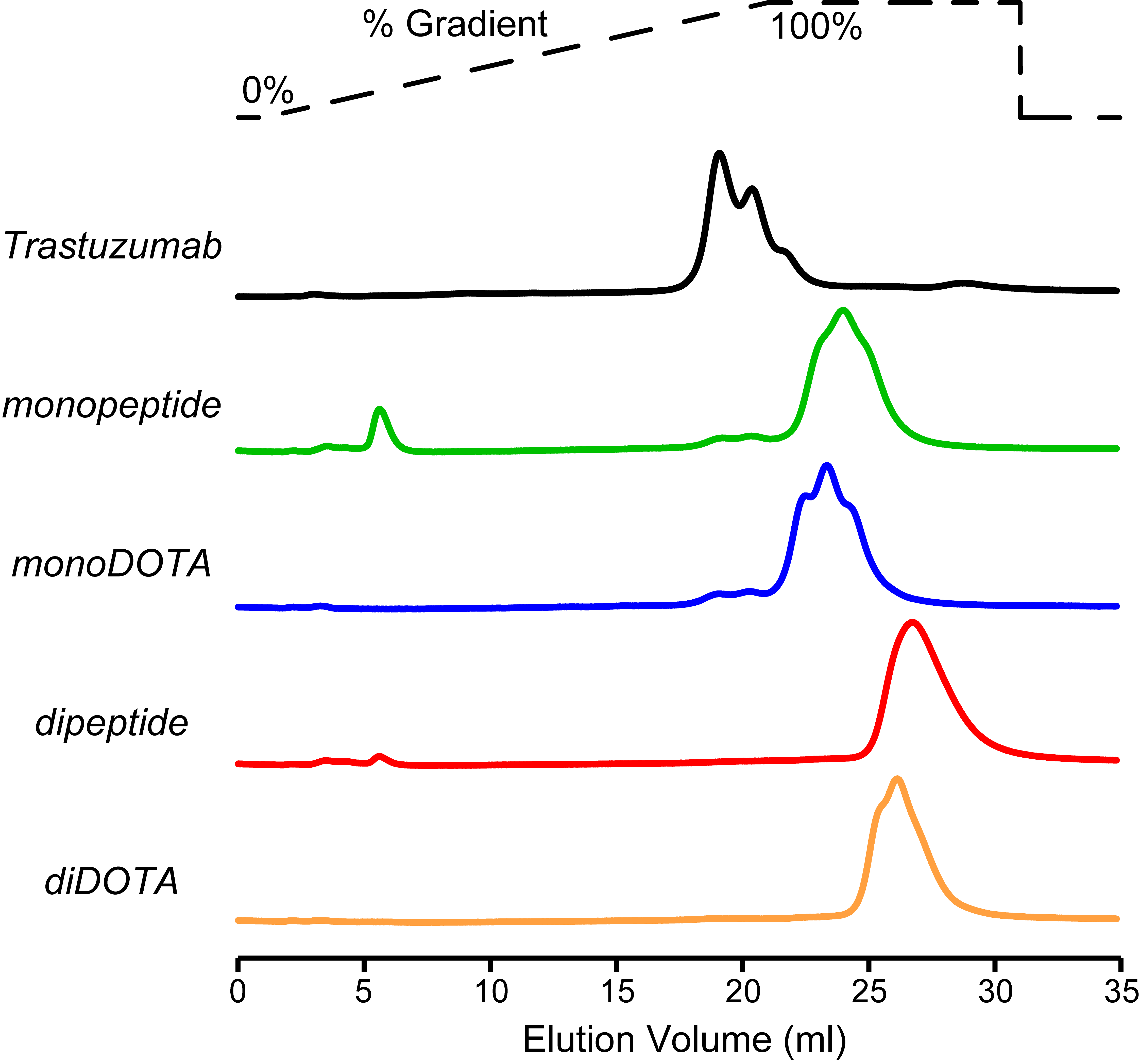


Fig. S2. FcγRIIIa column chromatography profiles of peptide/DOTA-conjugated trastuzumab.

An engineered FcγRIIIa column chromatography profiles. Non-glycosylated, mutated and recombinant human FcγRIIIa column was developed by TOSOH. Absorbance at 280 nm was monitored. The gradient profile (0% to 100% in 20 mL) is shown on top (dashed line). As the elution gradient increases, the more strongly bound antibody elutes.


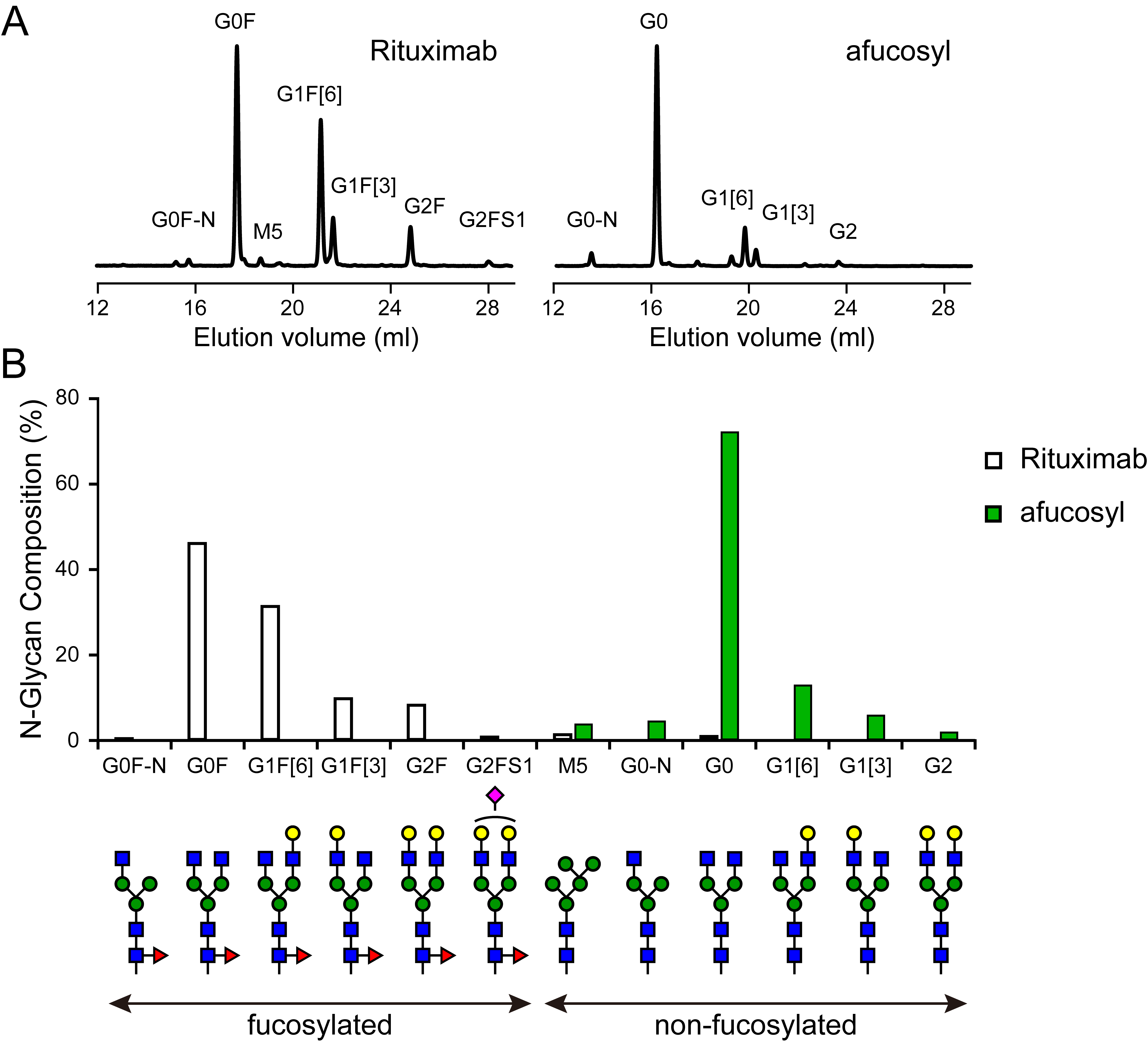


Fig. S3. N-Glycan profiles of rituximab and afucosyl rituximab.

(A) HILIC fluorescence chromatogram of 2-AB labeled N-glycans released from rituximab (left) and afucosyl rituximab (right). (B) N-glycan composition of rituximab (white bar) and afucosyl rituximab (green bar). Nomenclature and structures of Fc N-glycans are displayed below: blue square, N-acetylglucosamine; green circle, mannose; yellow circle, galactose; red triangle, fucose; purple diamond, sialic acid.

**
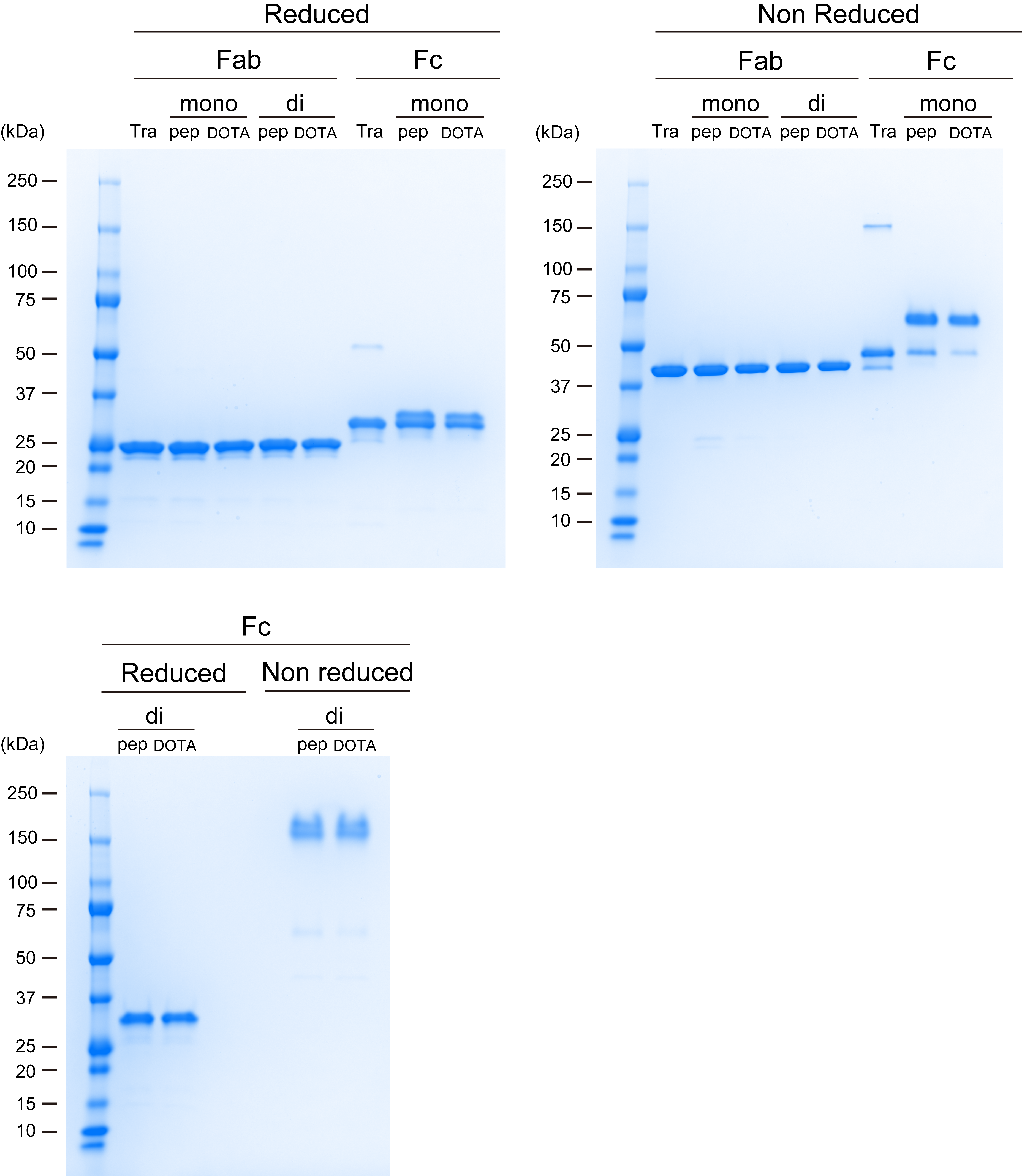
**

**Fig. S4.** SDS-PAGE analysis of purified Fab and Fc.

SDS-PAGE analysis of Fab and Fc. The samples were analyzed using 4–15% precast gel under non-reduced and reduced condition. Since the peptide contains positive charged restudies such as Lys and Arg, the bands of peptide/DOTA conjugated Fc appeared in the upper side (negative electrode side).

Table S1. Kinetic parameters of the FcγRIIIa binding.

|  | *k_on_* (M^-1^ s^-1^) (×10^5^) | *k_off_* (s^-1^) (×10^-3^) | *K_D_* (nM) |
| --- | --- | --- | --- |
| Trastuzumab | 5.5 | 28.1 | 51.0 |
| monopeptide | 11.1 | 11.6 | 10.4 |
| monoDOTA | 9.5 | 11.6 | 12.2 |
| dipeptide | 28.0 | 7.8 | 2.8 |
| diDOTA | 24.6 | 8.9 | 3.6 |
| Rituximab | 5.4 | 33.1 | 61.4 |
| dipeptide | 64.1 | 17.9 | 2.8 |
| afucosyl | 9.9 | 10.8 | 10.9 |
| afucosyl dipeptide | 25.1 | 2.7 | 1.1 |

Table S2. Summary of HDX-MS experiments.

| Data Set | Trastuzumab | monopeptide | monoDOTA | dipeptide | diDOTA |
| --- | --- | --- | --- | --- | --- |
| HDX time course (sec) | 60, 120, 240, 480, 960, 1920, 3840 | 60, 120, 240, 480, 960, 1920, 3840 | 60, 120, 240, 480, 960, 1920, 3840 | 60, 120, 240, 480, 960, 1920, 3840 | 60, 120, 240, 480, 960, 1920, 3840 |
| # of peptides | 143 | 145 | 146 | 150 | 141 |
| Sequence coverage | 79.4% | 80.2% | 80.2% | 80.2% | 80.2% |
| Average peptide length / Redundancy | 17.67 / 3.80 | 17.77 / 3.88 | 18.03 / 3.96 | 18.09 / 4.08 | 17.73 / 3.76 |
| Replicates | 1 | 1 | 1 | 1 | 1 |
| Repeatability (avg. stddev of #D) | 0.1412 | 0.1213 | 0.177 | 0.1189 | 0.1354 |

Table S3. DSC parameters of the purified Fab and Fc.

*Tonset*; thermal unfolding transition onset, Tm; thermal unfolding midpoint, *ΔH*; thermal unfolding enthalpy change, total area; total enthalpy change of thermal unfolding.

|  | *Tonset*  (°C) | *Tm₁*  (°C) | *∆H₁*  (kcal/mol) | *Tm₂*  (°C) | *∆H₂*  (kcal/mol) | Total Area  (kcal/mol) |
| --- | --- | --- | --- | --- | --- | --- |
| Trastuzumab Fab | 71.75 | 82.60 | 399 | - | - | 418 |
| monopeptide Fab | 73.23 | 82.99 | 427 | - | - | 445 |
| monoDOTA Fab | 74.12 | 82.98 | 429 | - | - | 444 |
| dipeptide Fab | 74.05 | 82.99 | 425 | - | - | 441 |
| diDOTA Fab | 73.48 | 82.96 | 443 | - | - | 465 |
| Trastuzumab Fc | 62.16 | 69.76 | 156 | 82.35 | 169 | 314 |
| monopeptide Fc | 63.61 | 76.13 | 166 | 83.87 | 198 | 353 |
| monoDOTA Fc | 68.16 | 76.66 | 143 | 84.12 | 250 | 381 |
| dipeptide Fc | 71.3 | 82.6 | 140 | 86.11 | 223 | 366 |
| diDOTA Fc | 78.36 | 83.83 | 90.3 | 87.73 | 356 | 437 |
